# Beauty through Hygiene—Common-sense Ways to Health for Girls

**Published:** 1904-11

**Authors:** 


BEAUTY THROUGH HYGIENE—COMMON-SENSE WAYS TO HEALTH FOR GIRLS. By Emma E. Walker, M.D. New York: A. S. Barnes & Co. 1904.

This work is a plea for a more natural existence among girls, and a more careful observance of the laws of health. Specific directions as to exercise, diet and the like are given, with recommendations for the care of the skin, general management of over-thinness and over-fatness, constipation and similar disorders. The book could be read by the doctor with profit, as it gives the answer to many questions that he is sure to be asked by mothers of young girls. Girl readers will be benefited by its teachings, which are clean, wholesome and healthful.

